# The Diagnosis of Melanotic Cancer by the Urine

**Published:** 1859-07

**Authors:** 


*The Diagnosis of Melanotic Cancer by the Urine.* By Dr. Eiselt. (Vierteljahrsschrift für die practische Heilkunde, xv. Jahrgang, 1858, Dritter Band.)

Dr. Eiselt reports three cases which appear to show that the urine offers a means of diagnosis in melanotic cancer. The first is that of a man, aged sixty, who, in 1856, came under observation, with symptoms of hepatic cancer, and with cancer of the left eye. There was no icterus, but the urine exhibited a remarkable peculiarity; when passed, it was perfectly clear; but on standing, it became as dark as porter, without losing its transparency. A portion was drawn by the catheter; it was found to contain copious uric acid, and a normal quantity of urea; when exposed to air and light, it became dark in a few hours; but concentrated nitric acid caused the change instantly; other oxidizing substances, especially chromic acid, produced the same effect; and the black matter was regarded as melanin, which induced the opinion, that the cancer was melanotic, a diagnosis confirmed by the autopsy. A year later, a man, aged sixty-eight, was admitted into the Prague Hospital, with cutaneous melanotic cancer. The urine, at first, exhibited the peculiarity shown in the last case feebly; but as the disease spread to the internal organs, and especially as the liver became affected, the reaction of the urine became as characteristic as in the former case. A third case occurred in May, 1858, in which there was hepatic cancer, and cancer of one eye. The urine again, induced the attending physician to diagnose melanotic cancer; some urine of May 8th was closed hermetically, and kept in the dark; some that was passed on May 9th, was also closed hermetically, and placed in the shade. On May 25th, Dr. Eiselt exhibited both specimens to the College of Physicians of Prague; the urine of May 8th was slightly turbid, pale yellow, and had deposited phosphates; the urine of May 9th was black with reflected, and dark-brown with transmitted light. On opening the first specimen, nitric acid and chromic acid at once induced the black color. The autopsy confirmed the diagnosis of melanotic cancer, for which there had been no other indication.

